# COVID-19 in Kyrgyzstan: Navigating a way out

**DOI:** 10.7189/jogh.11.03020

**Published:** 2021-01-30

**Authors:** Kenesh Dzushupov, Don Eliseo Lucero-Prisno, Dmitry Vishnyakov, Xu Lin, Attaullah Ahmadi

**Affiliations:** 1 Department of Public Health, International School of Medicine, Bishkek, Kyrgyzstan; 2 Department of Global Health and Development, London School of Hygiene and Tropical Medicine, London, United Kingdom; 3 Faculty of Management and Development Studies, University of the Philippines (Open University), Los Baños, Laguna, Philippines; 4 Department of Thoracic Surgery, The First Affiliated Hospital, School of Medicine, Zhejiang University, Hangzhou, Zhejiang, PR China; 5 Medical Research Center, Kateb University, Kabul, Afghanistan; 6 Global Health Focus Asia, Kabul, Afghanistan

The COVID-19 pandemic is the greatest global health crisis of our time [1]. Since the pandemic started in China, in December 2019, the disease has been creeping into almost every country across the globe [2,3]. The pandemic has led to a global cumulative incident of 47 901 761 confirmed cases, and 1 221 479 deaths by November 4, 2020 [4]. The first case of COVID-19 in Kyrgyzstan, a mountainous land-lock country in Central Asia, was detected on March 18, 2020 [5]. Thereafter the country was plagued with the pandemic and went through a lockdown, declaration of state of emergency, steady case increase and devastating peak of the wave with total 60 279 confirmed cases, 1159 deaths and 51 288 recoveries across the country by November 4, 2020 [4]. This paper aims to provide a commentary on the history and current state of the COVID-19 pandemic in Kyrgyzstan and the country’s efforts to address the threat.

The healthcare system of Kyrgyzstan has been going through various reforms for the last couple of decades under the supervision and support of largest bilateral donors and partners such as the World Bank, the Asian Development Bank, the World Health Organization, the European Union, and the Islamic Development Bank. These wide-ranging reforms undertaken were somewhat successful, mainly improving on the healthcare system of Kyrgyzstan making it relatively strong and with better health outcomes compared to other countries with a similar income level [6,7]. The healthcare expenditure of the country is 6.6% of GDP and the healthcare capacity shows having 45 hospital beds, 18.8 physicians, 64 nurses and midwives per 10 000 people [6]. Thus said, the emergency preparedness of Kyrgyzstan for the pandemic was expected to be above the average compared to neighbouring countries [7].

## FIRST CASES AND RESPONSES

Three citizens returning from a pilgrimage in Saudi Arabia were tested positive on March 18, 2020 [5]. Immediately after the first cases, Kyrgyzstan enforced containment measures to curb the virus spread by establishing checkpoints in each city, temporarily closing cafes, shopping malls, and other entertainment places, permitting only essential services such as grocery stores, food markets, pharmacies and medical centers on March 22, 2020 [5]. The large gatherings along with prayers at mosques and churches were banned. People were recommended to maintain a one-meter physical distance, refrain from physical contacts such as hand-shaking, and encouraged the wearing of face masks. The government closed country borders to foreigners and barred export of medicines and medical equipment as well as some food products and other essential goods [8].

## STATE OF EMERGENCY

Despite the strict measures, the numbers of COVID-19 cases were constantly increasing. The government declared a state of emergency on March 25, 2020 in the three major cities of Bishkek, Osh and Jalal-Abad. The locals were forbidden to get outside of their homes during curfew hours and were only allowed out for essential businesses such as buying food, medicines and visiting a medical facility during the day time. The government prohibited entering the regions with declared state of emergency except for citizens with local residence permits. All state employees and government servants had to work remotely from home except employees of emergency services. Schools and universities were closed and administrations of these institutions were recommended to continue operating using virtual means [9].

The state of emergency was terminated on May 10, 2020. Yet, quarantine measures were still in place in the biggest cities of Bishkek and Osh, and some highly affected districts of Kyrgyzstan [5]. The declaration of state of emergency allowed to delay dramatic uncontrolled spread of the virus around the country and gave valuable time to prepare healthcare system for possible increase of cases. Thus, the average number of daily COVID-19 incident cases did not exceed 100 until the middle of June 2020 [4].

## WAVE ON THE RISE

The Kyrgyz government lifted majority of quarantine restrictions after June 1, 2020. All business activities such as production and sales, consumer services, tourism and recreational activities were resumed [5]. At once, the number of incident cases of COVID 19 began increasing dramatically and by the end of the June 2020, the number of daily incident cases was almost 3 times higher compared to the start of the month [4]. Despite of all preparedness measures healthcare facilities started to experience difficulties with the increase of new cases prompting the Ministry of Health to develop an action algorithm, which included guidelines wherein individuals without symptoms and with the temperature not higher than 38 degrees Celsius be treated at home and not hospitalized [5].

**Figure:**
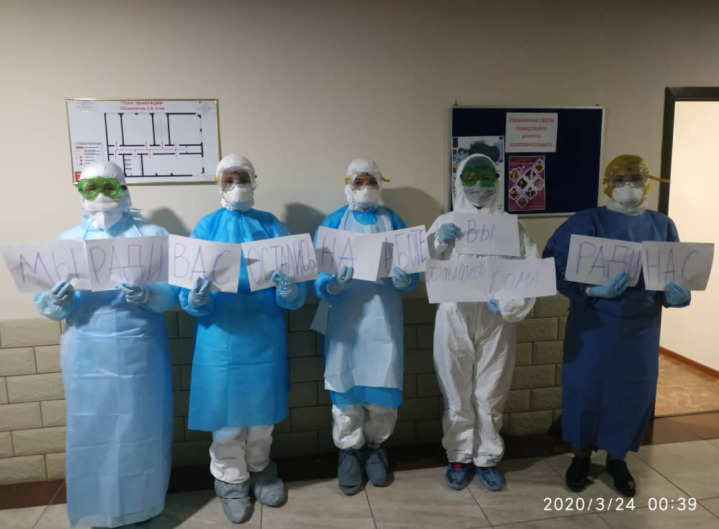
Photo: Health workers on the frontline of the fight against the COVID-19 pandemic in Kant town, Kyrgyzstan, saying “We stay at work for you, you stay home for us”. (Owned by one of the co-authors, used with permission).

## PEAK OF THE WAVE

As daily new cases continued to increase [4], the government ordered all private organizations and state bodies to return to teleworking. Authorities of Bishkek, the biggest city, imposed more restrictions on working hours of public transport, public places and city markets [9].

**Figure 1:**
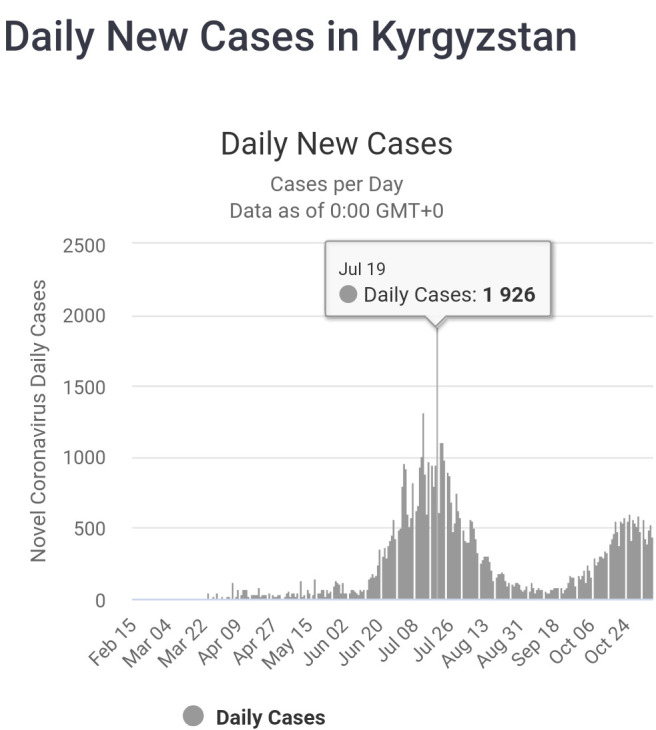
Novel coronavirus 19 (COVID-19) daily new cases in Kyrgyzstan.

In the middle of June 2020, the situation started to exacerbate dramatically. Bishkek with the highest number of cases, lack of medical staff, hospital beds and equipment, became the epicenter of the pandemic. Health authorities started to categorize all cases of acute pneumonia as COVID-19 cases. Previously, the statistics on death resulting from pneumonia and COVID-19 were classified separately, but due to the sharp increase in the number of hospitalizations from pneumonia, it was not feasible to make such a distinction [9]. The peak of the wave with 1926 cases on a single day was reached on July 19, 2020 (**Figure 1**) [4]. The struggle of the healthcare system by that period can be described by two facts: half of the ventilators were not functional and one in four recorded cases of COVID-19 were among health workers [9]. Furthermore, an overwhelmed healthcare system forced providers to focus more in combatting the pandemic thus resulting in limited access to healthcare of patients suffering from other diseases [5].

In order to cope with the emergency situation, the government opened departments for COVID-19 patients in several hospitals and some outpatient facilities in various districts of Bishkek. 100 health workers were recalled to the city from other regions, 30 Kyrgyz physicians returned from Moscow and many medical students were mobilized to fill the gap of healthcare workers. Moreover, 253 mobile teams were organized to provide support to homecare and conduct PCR test on COVID-19 using mobile laboratories [9]. All the country efforts in the fight against COVID paid off in the middle of August 2020 when the incidence started to decrease considerably. By the end of the month, it went down to 119 incident cases per day [4].

The latest statistics on COVID 19 accounted to a total of 60 279 confirmed cases, 1159 deaths and 51 288 recoveries across the country by November 4, 2020 [4]. Preliminary results of population-based sero-epidemiological on-going study showed about 30% seroprevalence of COVID-19 antibodies among Kyrgyz population and some Kyrgyz regions may account to be 62% in Narin and 50% in Bishkek of seropositivity among the general population [10].

The COVID 19 pandemic is the greatest challenge for Kyrgyzstan and the impact of the pandemic was devastating not only on health but also in socio-economic areas. Kyrgyzstan is a developing country and the national currency value heavily depends on exports of raw materials, gold and income of migrants working abroad. 22,4% of the population live below poverty line [7] and large number of people are self-employed and are not covered by social protection that official employees have [11]. The quarantine restrictions implemented by Kyrgyzstan and neighboring countries expect to lead to 4% drop in GDP by the end of 2020. Remittance inflows to Kyrgyzstan might sink by 25% constituting 33.23% of the country’s GDP in 2018 [7,12]. This is exacerbated by plummeting business activities in production textile and tourism [5]. Many financial international institutions lined-up to provide aid, but the scale and scope of the assistance will not suffice to compensate the disastrous economic impact in the long term [11].

## CONCLUSION

The devastating effect of the COVID-19 pandemic may hardly be overestimated in Kyrgyzstan. The country with a relatively strong healthcare system, cushioned the impact of the pandemic to a large extent by initiating precautionary measures, however, incident cases showed a steady increase every day and, finally, the pandemic overwhelmed the healthcare system and disrupted other health services. Though the pandemic is somewhat under control, as of this writing, and the population might have a form of indirect protection through herd immunity in some regions, the negative long-term effects of the pandemic are unavoidable. Thus, the country needs urgent measures which should include assessment of available resources in healthcare that might be mobilized for a possible second wave in some regions and the maintenance of balance in resource allocation between different health services. The government needs to employ caution when applying or lifting restriction policies to avoid sudden increase of cases and to balance it with the negative impact of restrictions that cause economic downturn. Major donors and regional powers need to give full support to Kyrgyzstan to strengthen its healthcare system because no one remains safe, if all is not safe.
